# The Microbiome-Mitochondria Dance in Prodromal Parkinson’s Disease

**DOI:** 10.3389/fphys.2018.00471

**Published:** 2018-05-09

**Authors:** Sandra M. Cardoso, Nuno Empadinhas

**Affiliations:** ^1^Center for Neuroscience and Cell Biology, University of Coimbra, Coimbra, Portugal; ^2^Institute of Cellular and Molecular Biology, Faculty of Medicine, University of Coimbra, Coimbra, Portugal

**Keywords:** mitochondria, bacteria, microbiome, neuronal innate immunity, Parkinson’s disease

## Abstract

The brain is an immunologically active organ where neurons and glia cells orchestrate complex innate immune responses against infections and injuries. Neuronal responses involve Toll-like or Nod-like receptors and the secretion of antimicrobial peptides and cytokines. The endosymbiotic theory for the evolutionary origin of mitochondria from primitive bacteria, suggests that they may have also retained the capacity to activate neuronal innate immunity. In fact, it was shown that mitochondrial damage-associated molecular patterns could signal and activate innate immunity and inflammation. Moreover, the mitochondrial cascade hypothesis for sporadic Parkinson’s disease (PD) argues that altered mitochondrial metabolism and function can drive neurodegeneration. Additionally, a neuroinflammatory signature with increased levels of pro-inflammatory mediators in PD affected brain areas was recently detected. Herein, we propose that a cascade of events initiating in a dysbiotic gut microbiome drive the production of toxins or antibiotics that target and damage mitochondria. This in turn activates neuronal innate immunity and triggers sterile inflammation phenomena that culminate in the neurodegenerative processes observed in the enteric and in the central nervous systems and that ultimately lead to Parkinson’s disease.

## Parkinson’S Disease Overview

Parkinson’s disease (PD), the most frequent neurodegenerative movement disorder, is characterized by severe loss of midbrain dopaminergic neurons in the SNpc and by the presence of intra-cytoplasmic inclusions of aggregated SNCA, known as LBs (Obeso et al., 2010). Sporadic PD (sPD) is a multifactorial disorder that evolves over decades without any motor complications. PD has a long prodromal phase during which several other symptoms develop, namely related to olfactory impairment, sleep disturbances, and depression (Reichmann et al., 2009). Another common underlying symptom described for the prodromal phase in PD patients is GI dysfunction that also includes dysphagia, gastroparesis, and severe constipation (Pfeiffer, 2003). These symptoms correlate with Braak staging whereas SNCA-immunopositive Lewy neurites and LBs target specific induction sites: initially in the dorsal motor nucleus of the glossopharyngeal and vagal nerves and in the anterior olfactory nucleus (Braak et al., 2003a).

Because LBs are also detected in the ENS of earliest and asymptomatic stage patients (Natale et al., 2011), the GI tract was proposed as an early target of PD pathology. Despite this, several evidences resulting from brain autopsy, animal models and cellular studies show that PD neurodegeneration involves multiple cellular processes, including mitochondrial dysfunction, oxidative stress, proteasomal and autophagic impairments and neuroinflammation (Olanow, 2007).

## Mitochondrial Involvement in Parkinson’S Disease Etiology

Mitochondria host biochemical reactions essential for normal cell functioning, namely energy production and maintenance of redox homeostasis (Lobet et al., 2015). Mitochondria were associated to sPD pathology when deficits in mitochondrial NADH dehydrogenase (complex I) activity were identified in the SNpc of post-mortem PD patient’s brains and in their platelets (Cardoso, 2011). Our group demonstrated that dysfunctional mitochondria from PD patients’ trigger several pathogenic features observed in PD subject brains, such as the generation of protein aggregates (LBs “like”) (Esteves et al., 2009), microtubule disassembly, disruption of intracellular trafficking (Esteves et al., 2010) and accumulation of autophagosomes and autophagic substrates (Arduíno et al., 2012).

The complex I inhibitors 1-methyl-4-phenyl-1,2,3,6-tetrahydropyridine and rotenone are widely used as *in vitro* and *in vivo* models of PD given that they recapitulate the main features of the disease (Meredith and Rademacher, 2011; Xiong et al., 2012). Mitochondrial dysfunction in PD tissues and models is also characterized by a decrease in mitochondrial membrane potential (Mann et al., 1992; Esteves et al., 2008), and by an increase in mitochondrial pool fragmentation and cristae disruption (Baloyannis et al., 2006; Santos et al., 2015). Accordingly, at a functional level, brain bioenergetics is compromised in PD where PET scans show decreased glucose utilization in PD individuals in the occipital cortex compared to control individuals (Schapira, 2008).

## Relevance of a Bacterial Origin of Mitochondria

After exposure to a new pathogen, our innate immune system protects us from infection. Innate immune responses are not specific to a particular pathogen and depend on the recognition of several conserved features of pathogens (Ward and Rosenthal, 2014). The innate immune response relies on PRRs to identify PAMPs, many of which are normal components of bacterial cells (Pallen, 2011).

Mitochondria share a common ancestor with Alphaproteobacteria and so proposed to be derived from ancestral bacterial endosymbiosis. The evidence supports a common origin for mitochondria and bacteria related to the *Rickettsiales* that have extremely reduced genomes and have obligate intracellular lifestyles (Fitzpatrick et al., 2006). mtDNA shares features with the genome of *Rickettsia prowazekii* but the similarities between bacteria and mitochondria extend beyond the abundance in the distinctive lipid cardiolipin in the inner membrane, to the numerous small molecule transport systems and to an electron transport chain that pumps protons across the inner mitochondrial membrane with the resulting proton motive force driving ATP synthesis via the F_1_ ATP synthase. Additionally, both the matrix of mitochondria and the cytosol of bacteria contain DNA, tRNA, ribosomes, and numerous soluble enzymes; both reproduce by binary fission and bear a *N*-formylmethionine start residue in their proteins. Remarkably, some bacterial PAMPs persist in mitochondria, such as formyl peptides that activate FPRs and unmethylated CpG dinucleotides, which activate TLR. Therefore the innate immune system does indeed recognize mitochondrial bacterial motifs, also called DAMPs. Upon mitochondrial release of DAMPs a sterile inflammation is activated that mimics the response to infection (Pallen, 2011).

## Mitochondria Damps Triggers Innate Immunity

The innate immune response can also be triggered by tissue damage independently of infection, a process also referred to as sterile inflammation, during which damaged cells release endogenous messengers known as DAMPs that are able to activate TLRs (Wilkins et al., 2017). At least 13 mammalian TLR isoforms are known, and each is capable of recognizing certain types of PAMPs or DAMPs (Takeuchi and Akira, 2010). Since mitochondria are an important source of DAMPs, the release of these mitochondrial DAMPs upon injury activates the innate immune system (Taanman, 1999). mtDNA is similar to bacterial DNA containing CpG motifs, which activate the TLR9 (Taanman, 1999; Zhang et al., 2010). Moreover, mitochondrial protein synthesis is initiated with the residue *N*-formylmethionine, similar to bacterial protein synthesis (Rabiet et al., 2007). The resulting bacterial *N*-formylated peptides are known to act as PAMPs by binding and activating G protein-coupled FPRs (Gurung et al., 2015), while the mitochondrial *N*-formylated peptides act as DAMPs through activation of the FPR1 (Taanman, 1999). Several studies have now described a crucial role for mitochondria in the regulation and activation of NLR specifically the Nlrp3 inflammasome (Latz et al., 2013). The inflammasomes are intracellular molecular platforms activated upon cellular infection or sterile stressors, which activate the pro-inflammatory cytokines interleukin-1β (IL-1β) and IL-18, to trigger cell death (reviewed in Zhou et al., 2011). A variety of insults, resulting from cellular infection or stress, can promote mitochondrial dysfunction and activate the Nlrp3 inflammasome (Schroder and Tschopp, 2010; Latz et al., 2013). While initial studies showed that mitochondrial dysfunction and mtROS production are necessary for Nlrp3 inflammasome activation (Nakahira et al., 2011; Shimada et al., 2012), further evidence has shown that mtDNA translo cation to the cytosol plays an active role in this process, where it can directly bind to and activate the Nlrp3 inflammasome (de Andrade Rosa et al., 2006). In addition, the mitochondrial lipid cardiolipin is also essential for Nlrp3 inflammasome activation, by directly binding to Nlrp3, downstream of mitochondrial dysfunction (Iyer et al., 2013). Altogether, by sensing mitochondrial DAMPs, the Nlrp3 inflammasome plays a critical role in integrating mitochondrial dysfunction in a pro-inflammatory signaling response, thus explaining the association of mitochondrial damage with inflammatory diseases.

Despite the great number of studies describing mitochondria as a source of DAMPs, the potential for mitochondrial DAMPs to trigger, or exacerbate, inflammation in the brain is now being explored. In recent studies, this potential was tested by treating different brain cell types with mitochondrial components, and measuring markers of inflammation. Neuronal and microglial cell lines exposed to mitochondrial lysates displayed increased markers of inflammation, with mtDNA being identified as the candidate DAMP responsible for the inflammatory changes (Wilkins et al., 2015). Recently, it was observed that stereotactic injection of mitochondrial lysates or purified mtDNA into animal hippocampi induced pro-inflammatory modifications (Wilkins et al., 2016), such as increased levels of hippocampal TNFα mRNA, glial fibrillary acidic protein and NFκB phosphorylation in the cortex.

## Neuronal Innate Immunity Activation in Parkinson’S Disease

Innate immunity reacts to different insults that may challenge the integrity of the CNS. This process is initiated by receptors of the TLR family that are activated by PAMPs or DAMPs. In the brain, this response is considered to be mediated by microglial cells, the major antigen-presenting cells in the CNS. Nevertheless, neurons also express critical TIR domain-containing adaptors that transduce signals of TLR, namely TLR1, TLR2, TLR3, TLR4, TLR7, and TLR9, and regulate the expression of various cytokines (Liu et al., 2014). Indeed, TLR3 and 7, localized in the neuronal endosomal compartment, play a role in neurite outgrowth. In neurons, TLR9 mainly found in the ER (Shintani et al., 2014), reduces the calcium transfer to the mitochondria promoting autophagy and cell survival (de Bernard and Rizzuto, 2014; Shintani et al., 2014). Others also found that TLR4 signaling regulates axonal growth, neuronal plasticity and even adult neurogenesis (Okun et al., 2011). Moreover, *in vitro* activation of neuronal TLR4 by LPS induces a strong expression of neuronal chemokines. These data revealed that neuronal TLR4 activation may play a central role in the onset of innate immunity during CNS infection or harm (Leow-Dyke et al., 2012). It is assumed that the cytokines produced by neurons may be just enough to recruit and activate local microglia without causing global brain inflammation. So it is perceived that also neuronal cells are able to mount an innate immune response. In fact, CNS neurons can be crucial sensors of infection since they respond to LPS by producing pro-inflammatory chemokines that in turn lead to activation of endothelial cells (Leow-Dyke et al., 2012). Interestingly, also ENS neurons respond to LPS and produce TNF-α (Coquenlorge et al., 2014). Regardless of PD being characterized by a slow and progressive degeneration of dopaminergic neurons in the SNpc, the cause of this neuronal loss is still poorly understood. Most relevant is the possibility that genetically determined age-dependent decline in mitochondrial function of the PD-typical pathologic cascade, gut bacteria or even their metabolites targeting the mitochondria, could activate innate immunity in dopaminergic neurons, due to the exposure of DAMPs, and in this way contribute to low-grade inflammation.

It was shown in PD cellular and animal models that mitochondrial network is highly fragmented. Mitochondrial fission is a prerequisite for the selective targeting of dysfunctional mitochondria for degradation by the lysosome in a process called mitophagy (Santos et al., 2015; Esteves et al., 2018). Nevertheless, it was recently proven that mitochondrial fission leads to the exposure of the inner membrane phospholipid, cardiolipin, which serves an important defensive function for the elimination of damaged mitochondria (Chu et al., 2013). Since cardiolipin is only found in mitochondrial and bacterial membranes it is considered a mitochondrial-derived DAMP that is detected by the Nlrp3 (He et al., 2016). NLR and TLR activation trigger the production of pro-inflammatory cytokines and AMPs (Lampron et al., 2013). Recently, it was also demonstrated that PD-associated SNCA proteins might be involved in the innate immunity response (Stolzenberg et al., 2017). It was proven that SNCA production mobilizes immune defenses against pathogens and the levels of mRNA of inflammatory cytokines in colonic biopsies from PD patients correlates with disease duration (Devos et al., 2013). Moreover, it was described that SNCA inserts in the mitochondrial membrane leading to mitochondrial dysfunction and also potentiate its fragmentation (Shen et al., 2014). These results seem to point to a positive feedback loop whereas mitochondrial dysfunction increases cardiolipin exposure, which in turn activates neuronal innate immunity. The question still remains on what is the role of SNCA under these conditions, one possibility being its involvement in the innate immunity pathway culminating in the potentiation of mitochondrial dysfunction.

## Mitochondria: Targets of the Human Microbiome

**FIGURE 1:**
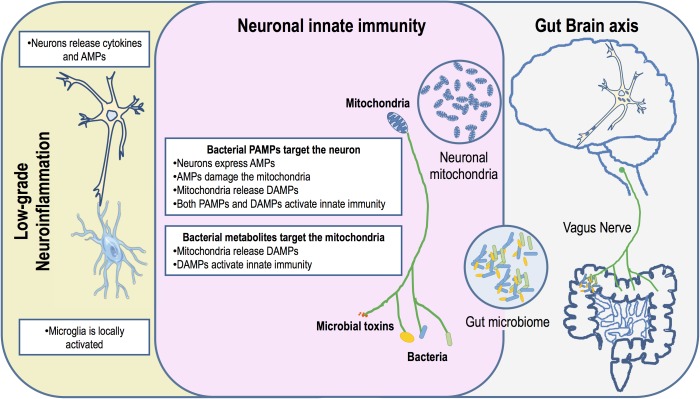
Schematic diagram indicating that neuronal mitochondria are primary gut bacteria targets. A dysbiotic gut harbors an inflammatory microbiota that could potentiate the production of microbial toxins. Either bacteria or bacterial toxins could activate innate immunity in the ENS and CNS through the vagus nerve, the gut-brain axis. Neuronal innate immunity is triggered by bacterial PAMPs or due to mitochondrial DAMPs. Mitochondrial damage may occur through the action of AMPs produced by the neuron as an arm of innate immunity activation or by the action of antibiotics produced by bacteria. PAMPs and mitochondria DAMPs activate the NLRs and TLRs leading to neuronal production of cytokines. These pro-inflammatory cytokines are released and activate low-grade inflammation through microglia. This chronic inflammation impacts neurons exacerbating AMPs production and mitochondrial damage.

Mitochondria play a key in the regulation of many immune functions through metabolic control, calcium homeostasis and ROS production, thus assisting host defenses against pathogens (Lobet et al., 2015). However, many bacteria have evolved several different types of effectors and mechanisms that target the mitochondria precisely as a strategy to circumvent mitochondria-dependent surveillance. In light of the endosymbiotic theory it can be argued that these mechanisms may have evolved in the ancestor bacteria as a strategy to gain competitive advantage in overpopulated environments. The mammalian gut harbors highly complex microbial communities with constantly balanced microbe–microbe and microbe–host interactions involving cooperative and competitive mechanisms to maximize the available resources and a shared co-existence (Sana et al., 2017). Some microbial species take advantage of nutrients produced by others in the community, while others target and kill their competitors by releasing toxic metabolites or by secreting effectors. We propose that gut dysbiosis increasingly associated to PD may, in the complex and competitive gut ecosystem, promote unrestrained and chronic production of microbial toxins that also target the mitochondria of ENS and CNS neurons (**Figure 1**). Indeed, PD biopsy studies confirmed the presence of LBs in organs innervated by the vagus nerve (Cersosimo et al., 2013), indicating this as the obvious route for disease progression between the gut and the brain. This led to the hypothesis that an exogenous toxin or pathogen can trigger the disease and spread via retrograde axonal vagus transport from the ENS to the CNS, and that the GI symptoms in the vast majority of PD patients are pre-motor manifestations of the disease (Braak et al., 2003a,b; Hawkes et al., 2007). Indeed, full truncal vagotomy significantly decreased the risk for subsequent PD, which suggests that the vagus nerve is indeed a key player in PD pathogenesis, again corroborating the involvement of an enteric pathogen or toxin in disease progression (Svensson et al., 2015). Recently, the development of SNCA pathology in genetically susceptible mice was shown to require the presence of gut microbiota, as evidenced by the limited pathophysiology observed in germ-free and antibiotic-treated susceptible mice, although the effect of antibiotics on the mitochondria was not evaluated. Remarkably, administration of certain microbial metabolites to genetically modified germ free mice reproduced major features of the disease, comparable to PD induced in mice with a complex microbiota (Sampson et al., 2016). Although PD gut microbiota signatures have recently begun to emerge, their functional interpretation still remains largely elusive (Hill-Burns et al., 2017; Scheperjans, 2018).

Many toxins produced by eventual gut microbes can damage mitochondria. Some *Clostridium difficile* strains secrete toxins that inhibit the mitochondrial ATP-sensitive potassium channels, drive mitochondrial membrane hyper-polarization, apoptosis and disruption of the gut epithelial barrier (Matarrese et al., 2007). *Vibrio cholera* secretes a toxin (VopE) that inhibits mitochondrial network reorganization (Suzuki et al., 2014). Although these toxins have not been identified in commensal microbiota, gene clusters for their biosynthesis may be present and drive the synthesis of related toxic compounds that may impact mitochondria. Hypotheses for the etiology of PD pathology based on chronic exposure to environmental toxins have been proposed as the underlying cause for PD onset (Tanner et al., 2011). In theory, the gut microbiota might also represent the “environmental” source of toxins to which the host would be exposed. In addition to the above examples of toxins produced by pathogenic gut microbiota, low molecular weight antibiotics of different classes are also known to induce mitochondrial dysfunction and oxidative damage with pathological consequences (Kalghatgi et al., 2013). Some classes of antibiotics target the bacterial protein synthesis machinery and inadvertently also the mitochondrial ribosomes (mitoribosomes) with potentially severe side effects in the host (Hobbie et al., 2008; Qian and Guan, 2009). The mitochondrial protein synthesis apparatus is similar to that of bacteria as a result of a shared origin and later endosymbiosis. Consequently, mitochondrial ribosomes are frequently unintended off-targets of antibiotics such as the aminoglycosides directed to bacterial ribosomes (Hobbie et al., 2008). Actually, sensitivity to a given antibiotic is likely a multifactorial trait but the genetic makeup of sensitive individuals, including the observed higher mutation rates in mtDNA accumulated as a consequence of aging, may also be a major contributing factor (Qian and Guan, 2009; Pacheu-Grau et al., 2013). Considering that most of the known antibiotics in use since the 1940’s are of microbial origin and more prominently produced by members of the phyla Actinobacteria and Firmicutes as well as by some Fungi, is it reasonable to anticipate that some members of the gut microbiota may possess the genetic resources to synthesize other antibiotics or antimicrobials including toxins that may target their distantly related counterparts, the mitochondria. Indeed, unknown antimicrobials remain hidden in the largely unexplored human microbiome (Donia et al., 2014). Although their identity and effects in mitochondria with possible damage eventually leading to activation of innate immunity have not been addressed, the enormous biosynthetic potential for metabolites impacting microbes clearly indicates that we haven’t seen but the tip of the mediators regulating the complex microbial interactions within us, and which might foster mitochondrial damage and neurodegenerative processes.

## Author Contributions

All authors listed have made a substantial, direct and intellectual contribution to the work, and approved it for publication.

## Conflict of Interest Statement

The authors declare that the research was conducted in the absence of any commercial or financial relationships that could be construed as a potential conflict of interest.

## Abbreviations

AMP: antimicrobial peptides
CNS: central nervous system
DAMP: damage-associated molecular pattern
ENS: enteric nervous system
FPR: formyl peptide receptors
GI: gastrointestinal
IL: interleukin
LBs: Lewy bodies
LPS: lipopolysaccharide
mtDNA: mitochondrial DNA
mtROS: mitochondrial produced reactive oxygen species
NLR: NOD-like receptors
Nlrp3: NLR family pyrin domain containing 3
NOD: nucleotide-binding oligomerization domain-like
PAMP: pathogen-associated molecular pattern
PD: Parkinson’s disease
PET: positron emission tomography
PRR: pattern recognition receptor
SNCA: alpha-synuclein
SNpc: substantia nigra pars compact
TIR: Toll/interleukin-1 receptor
TLRs: Toll-like receptors
TNFα: tumor necrosis factor α
